# The prognostic value of the ratio of standard uptake value of lymph node to primary tumor before treatment of locally advanced nasopharyngeal carcinoma

**DOI:** 10.1007/s00405-022-07562-w

**Published:** 2022-08-06

**Authors:** Yunlong Lou, Dandan Chen, Zheng Lin, Jianda Sun, Li Song, Wenzhong Chen, Ming Zhang, Yibiao Chen

**Affiliations:** 1 Department of Nuclear Medicine, Meizhou People’s Hospital, Meizhou Academy of Medical Sciences Meizhou, Meizhou, China; 2 Department of Radiotherapy, Meizhou People’s Hospital, Meizhou Academy of Medical Sciences Meizhou, Meizhou, China

**Keywords:** Nasopharyngeal carcinoma, Tomography, Positron emission type, Standard uptake value, Deoxyglucose, Prognosis, Ratio

## Abstract

**Background:**

To evaluate the prognostic value of the ratio of the standard uptake value of the lymph node and primary tumor before the treatment of locally advanced nasopharyngeal carcinoma and examine the prognostic value of the tumor metabolic parameters (SUVmax_,_ MTV, and TLG) of the lymph node and primary tumor of locally advanced nasopharyngeal carcinoma.

**Methods:**

A total of 180 patients with locally advanced nasopharyngeal carcinoma diagnosed pathologically from January 1, 2016 to December 31, 2018 were selected, and the MEDEX system was used to automatically delineate the SUVmax, MTV, and TLG of the lymph node metastases and nasopharyngeal carcinoma primary tumor. In addition, the ratio of LN-SUVmax (SUVmax of the lymph node metastases) to T-SUVmax (SUVmax of the nasopharyngeal carcinoma primary tumor) was calculated, and a ROC curve was drawn to obtain the best cut-off value. Kaplan–Meier and Cox regression models were used for survival and multivariate analyses, respectively.

**Results:**

The median follow-up period for participants was 32 (4–62) months. Univariate analysis showed that age (*P* = 0.013), LN-SUVmax (*P* = 0.001), LN-TLG (*P* = 0.007) and NTR (*P* = 0.001) were factors influencing the overall survival (OS). Factors affecting local progression-free survival (LPFS) were LN-SUVmax (*P* = 0.005), LN-TLG (*P* = 0.003) and NTR (*P* = 0.020), while clinical stage (*P* = 0.023), LN-SUVmax (*P* = 0.007), LN-TLG (*P* = 0.006), and NTR (*P* = 0.032) were factors affecting distant metastasis-free survival (DMFS). Multivariate analysis showed that NTR was an independent influencing factor of OS (HR 3.00, 95% CI 1.06–8.4, *P* = 0.038), LPFS (HR 3.08, 95% CI 1.27–7.50, *P* = 0.013), and DMFS (HR 1.84, 95% CI 0.99–3.42, *P* = 0.054). Taking OS as the main observation point, the best cut-off point of NTR was 0.95. Kaplan–Meier results showed that the 3-year OS (97.0% vs 85.4%, *χ*^2^ = 11.25, *P* = 0.001), 3-year LPFS (91.3% vs 82.1%, *χ*^2^ = 4.035, *P* = 0.045), and 3-year DMFS (92.3% vs 87.9%, *χ*^2^ = 4.576, *P* = 0.032) of patients with NTR < 0.95 were higher than those with NTR > 0.95.

**Conclusions:**

High NTR before treatment indicates a poor prognosis for patients with nasopharyngeal carcinoma. This can serve as a reference value for the reasonable treatment and prognosis monitoring of such patients.

## Background

Nasopharyngeal carcinoma has a high incidence in southern China. Also called Cantonese cancer, it is the most common type of head and neck cancer. However, its cause, treatment, and prognosis are different from other head and neck tumors; the incidence of cervical lymph node and/or distant metastasis is high [1–3]. The high sensitivity of nasopharyngeal carcinoma to radiotherapy and the patient’s relatively young age of onset lead to a good overall prognosis. The use of concurrent radiotherapy and chemotherapy for nasopharyngeal carcinoma can achieve a 5-year overall survival (OS) rate and disease-free survival rate of up to 70% [4]. However, some nasopharyngeal carcinomas relapse or metastasize after treatment, making individualized treatment plans critical. Identifying prognostic factors that predict treatment outcomes more accurately may help patients who would benefit from more aggressive treatments. ^18^F-FDG-PET/CT has been widely used in the diagnosis and staging of patients with nasopharyngeal carcinoma. Many studies have also shown that SUV can be used for risk stratification and has good prognostic value [5]. Recently, some studies have shown that the SUV ratio (NTR) of the lymph node to the primary tumor is strongly associated with survival. Hung et al. [6] reported that NTR is an easily available indicator and has a good predictive value for distant metastasis after treatment, which may help determine more personalized treatments or designs for future clinical trials. In the literature, only one study [6] reported on the value of NTR in the survival and prognosis of patients with nasopharyngeal carcinoma. Our study aimed to further examine the prognostic value of NTR in advanced nasopharyngeal carcinoma.

## Material and methods

### Inclusion criteria

Pathologically diagnosed patients with nasopharyngeal carcinoma; patients who underwent a ^18^F-FDG-PET-CT examination before treatment and with complete PET-CT data and clinical data; patients with at least one neck lymph node metastasis and patients without distant metastasis.

### Clinical data

**Fig. 1:**
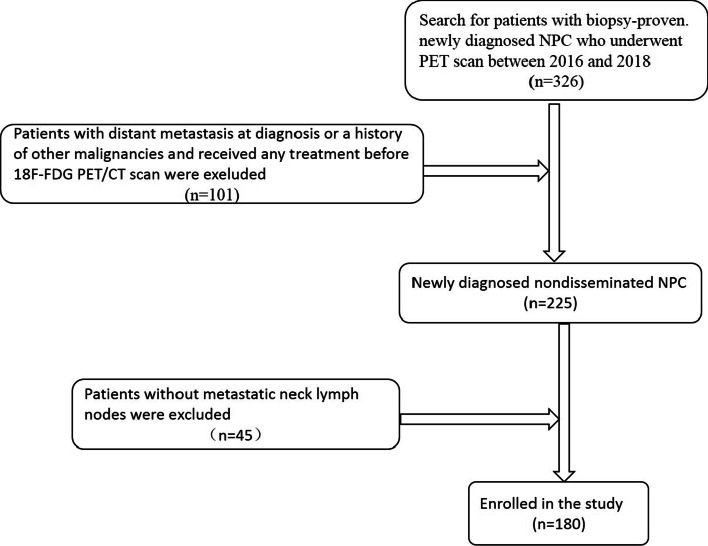
Flowchart of the patients with nasopharyngeal carcinoma

We performed a retrospective analysis of 225 patients with nasopharyngeal carcinoma. Among them, 45 patients without lymph node metastasis were excluded, and finally, 180 patients were included in the study [males: 121 (67.2%); females: 59 (32.8%)] (Fig. 1). The age of the patients was 16–82 years (median age 49.5 years). The pathological types of 180 patients were undifferentiated and differentiated non-keratinizing carcinoma. According to the 2017 UICC or American Joint Committee on Cancer (AJCC) version 8 of the nasopharyngeal carcinoma staging system, there were 27 patients in the T1 stage, 51 in the T2 stage, 61 in the T3 stage, and 41 in the T4 stage and 64 in N1 stage, 73 in N2 stage, and 43 in N3 stage. There were 24 clinical stages in stage II and 78 in stages III and IV.

### Follow-up

Outpatient and telephone follow-up periods were from the date of diagnosis to April 21, 2021, and the median follow-up time is 32 (4–62) months. After treatment, the frequency of follow-up for the first 3 years was once every 3 months and then every 6 months until the end of the follow-up period. Follow-up included routine head and neck examination, nasopharyngeal endoscopy, chest radiograph, and abdominal B-ultrasound. Further examinations were conducted to confirm the diagnosis in case of suspicious problems. The location and time of tumor residue, recurrence, and metastasis were recorded, and rescue treatments (such as re-radiation, surgery, and chemotherapy) were determined according to the severity of the disease. OS was defined as the time from the start of treatment to the death of the patient (for any reason). Local progression-free survival (LPFS) was defined as the time from diagnosis by pathology to the first discovery of local recurrence after treatment or to the last follow-up date. Distant metastasis-free survival (DMFS) was defined as the time from the start of treatment until the time when a patient had distant metastasis or the time to the last follow-up date.

### Treatment

All patients received radiotherapy, comprising IGRT and VMAT. The chemotherapy regimen in concurrent radiotherapy and chemotherapy was platinum single drugs (cisplatin, nedaplatin, and carboplatin), and the neoadjuvant chemotherapy regimen or adjuvant chemotherapy regimen was a platinum combination regimen (paclitaxel combined with platinum, paclitaxel + fluorouracil + platinum, and fluorouracil combined with platinum).

### PET/CT imaging

Imaging instrument was Biograph mCT.s20 from Siemens, Germany (16 rows CT). ^18^F-FDG was provided by the Guangzhou Isotope Center of the Chinese Academy of Atomic Energy, with a radiochemical purity of 95%. Before the PET/CT examination, all patients fasted for more than 4 h. The blood glucose was measured before the examination and controlled to be less than 11 mmol/L (198 mg/dL). ^18^F-FDG 5.55 MBq/kg (0.15 mCi/kg) was injected intravenously according to body weight, and patients were rested for 50–60 min until PET/CT imaging. The scan range was from the upper middle of the thigh to the top of the skull. The CT scan parameters were as follows: voltage, 140 kV; current, 110 mA; tube single-turn rotation time, 0.5 s; and layer thickness, 3 mm. The PET scan duration was 3 min per bed, and the PET image attenuation was corrected based on the CT image.

### Image analysis

The PET and CT images are sent to the MMWP22510 workstation for image fusion and image processing analysis. The region of interest (ROI) was delineated automatically with the 40% threshold of SUVmax, and the optimal boundary of the tumor was determined by manually adjusting the axial position. The primary tumor and lymph node SUVmax, MTV, and TLG were automatically delineated and acquired by MEDEX software. The SUVmax in each ROI was determined using the whole-body attenuation corrected image. The MTV was defined as the total tumor volume and TLG was defined as the total lesion glycolysis (Mean SUV × MTV).The maximum SUV (SUVmax) was defined as the highest activity concentration per injected dose per body weight after correction for radioactive decay. The T‑SUVmax, T-MTV, and T-TLG were delineated and acquired from the primary tumor and the LN‑SUVmax, LN-MTV, and LN-TLG were defined according to the SUVmax of the highest neck lymph node metastasis. The metastatic lymph nodes were defined as having (1) a minimal short axial diameter of 6 mm or larger for retropharyngeal lymph nodes, 11 mm or larger for jugulodigastric lymph nodes, and 10 mm or larger for other neck lymph nodes; (2) the presence of necrosis or peripheral hyperenhancement; (3) the presence of ill-defined irregular margins; (4) groups of three or more lymph nodes or asymmetrically prominent lymph nodes along the drainage chain.

### Statistical analysis

SPSS software was used to draw the ROC curve and OS was used as the main observation point to obtain the boundary value of SUVmax, MTV, and TLG of nasopharyngeal carcinoma primary tumor and metastasis. Among them, the parameter value corresponding to the maximum Youden index was defined as the grouping boundary point. Youden index = (sensitivity + specificity) − 1. The Kaplan–Meier method was used to calculate OS, LPFS, and DMFS using SPSS 21.0 software, the log-rank method was used for testing and univariate prognostic analysis, and the Cox regression model was used for multivariate prognostic analysis. *P* < 0.05 indicated significant difference.

## Results

A total of 180 patients with nasopharyngeal carcinoma were included in the analysis. The median survival time of patients with OS, LPFS, and DMFS were 60 months, 55 months, and 55 months, respectively. The rates of 3-year OS, 3-year LPFS, and 3-year DMFS were 91%, 93%, and 91%, respectively. Among the 180 patients, 11 had fatal events, 13 had recurrence and metastasis, 2 had simple local recurrence in the nasopharynx, 5 had simple neck lymph node recurrence, 1 had nasopharyngeal and neck recurrence, and 2 had local recurrence accompanied by distant metastasis. Distant metastases occurred in 12 patients: 2 liver metastases, 8 lung metastases, 6 bone metastases, 1 mediastinal metastasis, and 1 abdominal lymph node metastasis.

Factors analyzed in the study were general clinical factors and PET-related parameters including age, gender, T stage, N stage, clinical stage, pathological type, T-SUVmax, LN-SUVmax, and NTR.

**Fig. 2:**
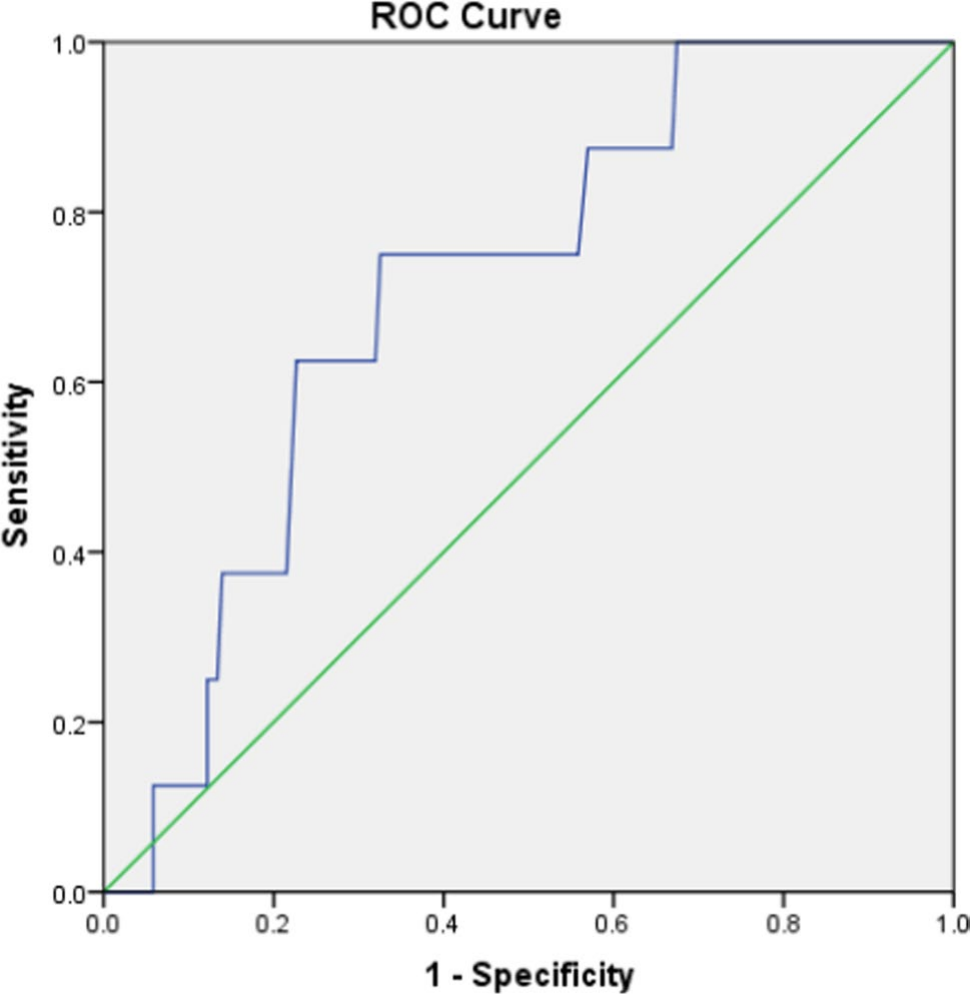
Receiver operating characteristic curve analysis of overall survival based on the NTR value. Area under the curve is 0.710 (*P* = 0.044, 95% confidence interval of 0.560–0.860), and the best cut-off value is 0.95

**Fig. 3:**
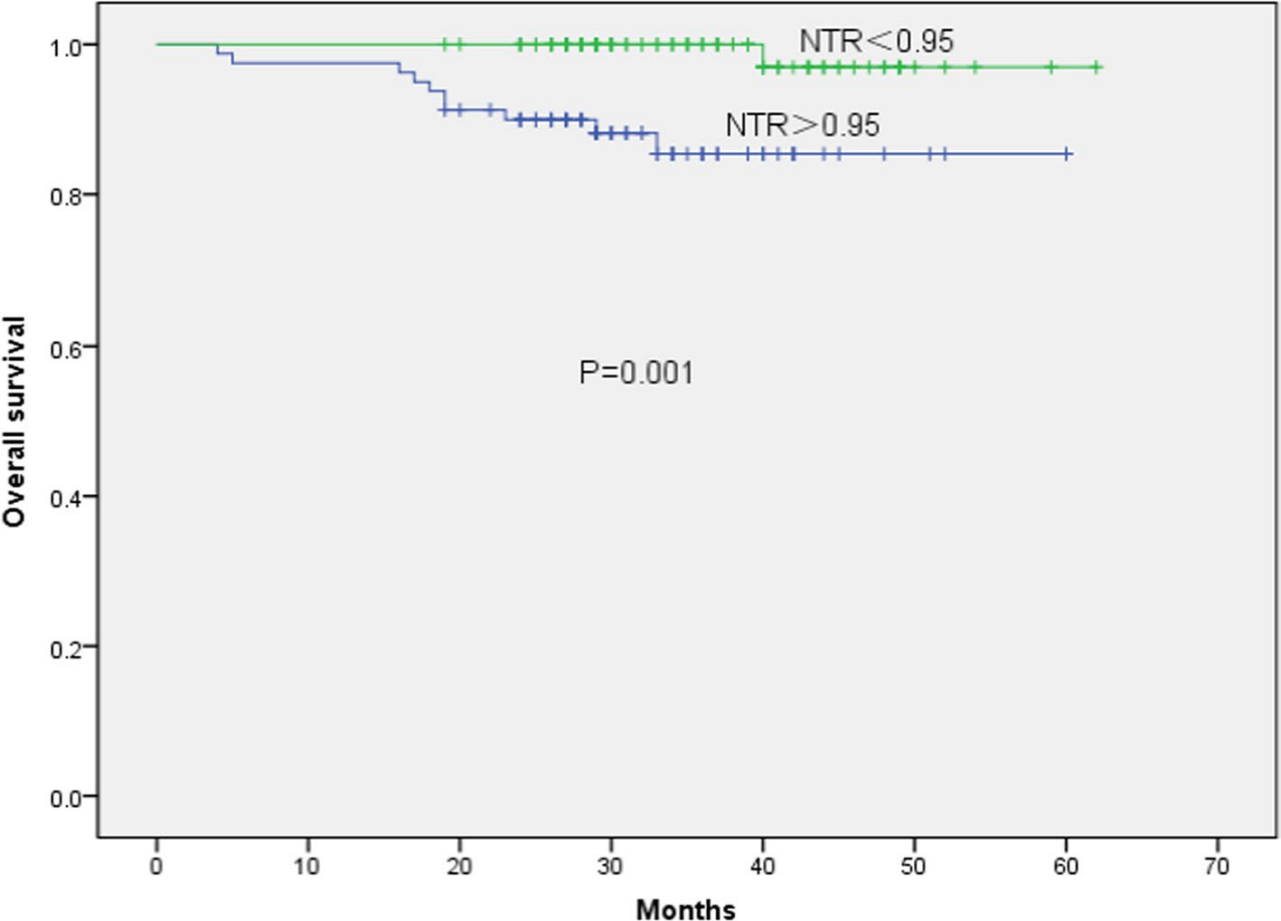
Kaplan–Meier survival curve of overall survival, stratified according to NTR, *P* = 0.001

**Fig. 4:**
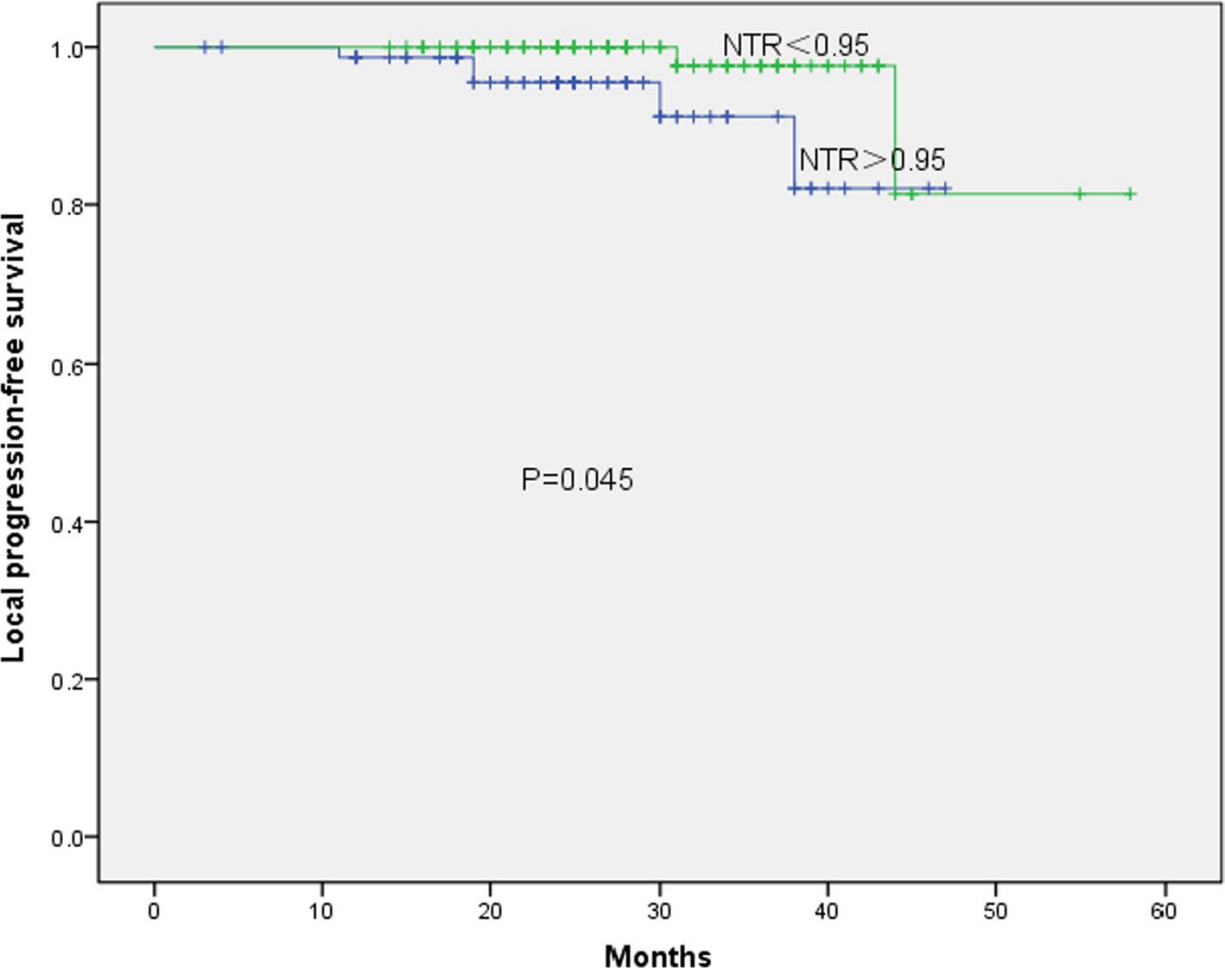
Kaplan–Meier survival curve of local progression-free survival, stratified according to NTR, *P* = 0.045

**Fig. 5:**
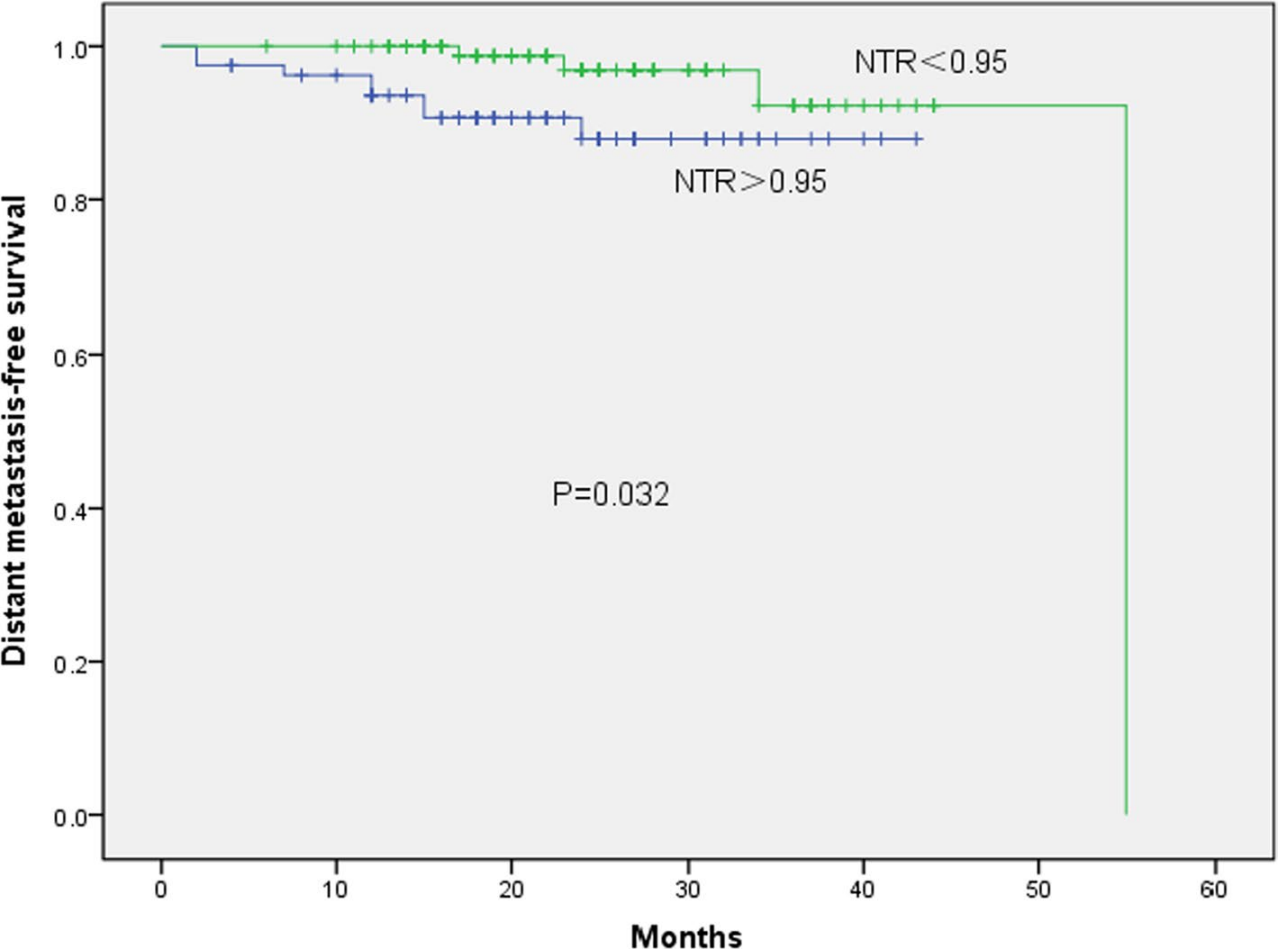
Kaplan–Meier survival curve of distant metastasis-free survival, stratified according to NTR, *P* = 0.032

Determination of the cut-off value of each parameter: with OS as the main survival end point, the boundary value of each parameter was determined according to the ROC curve. The best cut-off value of NTR related to OS was 0.95 (AUC of 0.710, *P* = 0.044, 95% CI 0.560–0.860, sensitivity = 0.750, and specificity = 0.674) (Fig. 2). The patients were divided into two groups: the high NTR group (NTR > 0.95; *n* = 80) and the low NTR group (NTR < 0.95; *n* = 100). The cut-off values of the other two parameters were T-SUVmax of 12.35 (AUC 0.582, *P* = 0.361, 95% CI 0.395–0.770, sensitivity = 0.636, specificity = 0.586) and LN-SUVmax of 9.32 (AUC 0.765, *P* = 0.003, 95% CI 0.650–0.879, sensitivity = 0.909, specificity = 0.521). A total of 93 patients had LN-SUVmax > 9.32 and 87 had LN-SUVmax ≤ 9.32.The results of univariate prognostic analysis of different survival endpoints are shown in Table 1. A univariate analysis of OS, LPFS, and DMFS for all patients was carried out. The observation targets were OS, LPFS, and DMFS. Univariate analysis showed that age, LN-SUVmax, LN-TLG, and NTR were associated with OS (*P* = 0.013, 0.001, 0.007, and 0.001, respectively); LN-SUVmax LN-TLG, and NTR with LPFS (*P* = 0.005, 0.003, and 0.020, respectively), and clinical stage, LN-SUVmax, LN-TLG, and NTR with DMFS (*P* = 0.023, 0.007, 0.006, and 0.032, respectively). Kaplan–Meier results showed that the 3-year OS (97.0% vs. 85.4%, *χ*^2^ = 11.25, *P* = 0.001), 3-year LPFS (91.3% vs 82.1%, *χ*^2^ = 4.035, *P* = 0.045), and 3-year DMFS (92.3% vs 87.9%, *χ*^2^ = 4.576, *P* = 0.032) of patients with NTR ≤ 0.95 were higher than those with NTR > 0.95 (Figs. 3, 4, 5). In addition, patients with LN-SUVmax < 9.32 had better 3-year OS (100% vs 88.0%, *χ*^2^ = 10.565, *P* = 0.001), 3-year LPFS (96.6% vs 93.4%, *χ*^2^ = 7.715, *P* = 0.005), and 3-year DMFS (97.8% vs 82.5%, *χ*^2^ = 7.387, *P* = 0.007) than patients with LN-SUVmax > 9.32.

**Table 1:** Univariate prognostic analysis of different survival endpoints in 180 patients with nasopharyngeal carcinoma

Variables	*N*	OS		LPFS		DMFS	
		*χ*^2^	*P* value	*χ*^2^	*P* value	*χ*^2^	*P* value
Sex		3.009	0.083	3.817	0.051	2.949	0.086
Male	121						
Female	59						
Age (year)		6.119	0.013	3.733	0.053	3.682	0.055
> 59.5	41						
≤ 59.5	139						
T stage		0.064	0.800	1.712	0.191	3.646	0.056
T1	27						
T2	51						
T3	61						
T4	41						
N stage		3.425	0.064	0.545	0.460	1.857	0.173
N1	64						
N2	73						
N3	43						
Clinical stage		3.011	0.083	0.758	0.384	5.175	0.023
II	24						
III	78						
IV	43						
Pathological type		0.088	0.767	0.088	0.767	0.049	0.824
UKC	179						
DNKC	1						
T-SUVmax		1.877	0.171	0.049	0.825	0.076	0.783
≤ 12.35	103						
> 12.35	77						
T-MTV		1.001	0.317	0.273	0.602	0.258	0.611
≤ 8.6	103						
> 8.6	77						
T-TLG		2.845	0.092	0.018	0.893	0.001	0.972
≤ 127.5	143						
> 127.5	37						
LN-SUVmax		10.565	**0.001**	7.715	**0.005**	7.387	**0.007**
≤ 9.32	87						
> 9.32	93						
LN-MTV		1.989	0.158	2.153	0.142	1.862	0.172
≤ 1.4	27						
> 1.4	153						
LN-TLG		7.252	0.007	8.893	0.003	7.480	0.006
≤ 20.4	91						
> 20.4	89						
NTR		11.25	**0.001**	5.385	**0.020**	4.576	**0.032**
≤ 0.95	100						
> 0.95	80						

Statistically significant indicators are shown in bold

*T-SUVmax* SUVmax of primary nasopharyngeal carcinoma, *T-MTV* MTV of primary nasopharyngeal carcinoma, *T-TLG *TLG of primary nasopharyngeal carcinoma, *LN-SUVmax* SUVmax of lymph node metastases, *LN-MTV* MTV of lymph node metastases, *LN-TLG* TLG of lymph node metastases, *NTR* ratio of LN-SUVmax to T-SUVmax, *UKC* undifferentiated keratinizing carcinoma, *DNKC* differentiated non-keratinizing carcinoma

Multivariate analysis showed that NTR was a significant influencing factor for OS (HR 3.00, 95% CI 1.06–8.48, *P* = 0.038) and LPFS (HR 3.08, 95% CI 1.27–7.50, *P* = 0.013), while only marginally significant for DMFS (HR 1.84, 95% CI 0.99–3.42, *P* = 0.054) Table 2. In addition, N stage (HR 3.32, 95% CI 1.04–10.66, *P* = 0.044) and clinical stage (HR 9.78, 95% CI 1.91–50.15, *P* = 0.006) were also influencing factors of LPFS, while clinical stage was a marginally significant influencing factor of DMFS (HR 2.12, 95% CI 0.99–4.55, *P* = 0.053).

**Table 2:** Cox multivariate analysis

	OS		LPFS		DMFS	
	HR(95%CI)	*P*	HR(95%CI)	*P*	HR(95%CI)	*P*
Sex Male vs female						
Age (year) > 59.5 vs < 59.5					1.74 (1.01–3.01)	0.046
T stage T1 + T2 vs T3 + T4						
N stage N1 + N2 vs N3			3.32 (1.04–10.66)	0.044		
Clinical stage II + III vs IV			9.78 (1.91–50.15)	0.006	2.12 (0.99–4.55)	0.053
T-SUVmax ≤ 12.35 vs > 12.35						
LN-SUVmax ≤ 9.32 vs > 9.32						
LN-TLG ≤ 20.4 vs > 20.4						
NTR < 0.95 vs > 0.95	3.00 (1.06–8.48)	0.038	3.08 (1.27–7.50)	0.013	1.84 (0.99–3.42)	0.054

*T-SUVmax* SUVmax of primary nasopharyngeal carcinoma, *LN-SUVmax* SUVmax of lymph node metastases, *LN-TLG* TLG of lymph node metastases, *NTR* ratio of LN-SUVmax to T-SUVmax, *HR* hazard ratio, *CI* confidence interval

## Discussion

The prognostic factors of malignant tumors have always been the focus of clinical attention because these help develop and determine more targeted treatments.Southern China has the highest incidence of nasopharyngeal carcinoma, with an annual incidence of 20–50 cases per 100,000 people. According to the 7th AJCC staging system, 60–70% of patients have locally advanced nasopharyngeal carcinoma (LA-NPC) [2, 3]. With the continuous development of radiotherapy techniques such as intensity-modulated radiotherapy and concurrent radiotherapy and chemotherapy, the regional control rate of nasopharyngeal carcinoma reaches 90%, while distant metastasis is the main reason for treatment failure. Therefore, to improve the prognosis and develop personalized treatments, patients with advanced nasopharyngeal carcinoma should be classified according to the risk of metastasis [5, 7, 8]. Recent studies have shown that MTV and TLG are better than SUV alone in predicting prognosis. Compared with MTV or SUV, TLG is expected to provide better prognostic stratification because it theoretically integrates tumor volume and glucose metabolism. Wang et al. [9] found that SUVmax and TLG are the influencing factors of 3-year LRFS and 3-year FFS through single-factor analysis, while SUVmax is the only independent influencing factor through multifactor analysis. Sdeng et al. [10] found that PET-related parameters are related to the prognosis of patients with nasopharyngeal carcinoma, among which MTV has a strong correlation with OS, EFS, and LRC. Yoon et al. [11] found that among the metabolic parameters of PET, TLG is the most useful prognostic factor for PFS, OS, LRFFS, and DFFS, and these results are consistent with the finding of Moon et al. [12], which showed that TLG of the primary tumor is a good metabolic prognostic factor in patients with nasopharyngeal carcinoma undergoing concurrent radiotherapy and chemotherapy. Jin et al. [13, 14] believed that SUVmax-N is an independent prognostic factor in determining patients with locally advanced nasopharyngeal carcinoma. Furthermore, combining SUVmax-N with clinical staging could influence the level of risk of locally advanced patients, thereby changing treatment decisions, and improving patient survival. In accordance with those results, our study showed that both the SUVmax and TLG of lymph node metastases were prognostic factors of OS, LPFS, and DFFS in univariate analysis, but it was not an independent factor in multivariate analysis. In contrast to those findings, our study found that the SUVmax, MTV, and TLG of primary nasopharyngeal carcinoma and the MTV of lymph node metastases neither were all prognostic factors. The discrepancy between our observations and those of previous studies might be due to different tumor stages, the different patient inclusion in our study, or that only patients with advanced disease stage were eligible for the present study.

T-SUVmax represents the highest voxel value within the volume of interest; however, it does not reveal heterogeneity in the tumor volumes. Xie et al. [15] found the most optimal cut-off value to be 8.0 for the SUVmax of the primary, although in multivariate analysis, it was not statistically significant in terms of the 5-year OS and PFS benefit. Lee et al. [16] also deemed a cut-off value of 8.0 to be optimum for prognosis prediction. In contrast, in the current study, the T-SUVmax cut-off of 12.35 did not have any major bearing on the OS, LPFS, and DFFS; however, SUVmax-N influenced the outcomes. Srinivas et al. [17] failed to identify a cut-off value for the pretherapy SUVmax that could predict the probable outcome of therapy.

Several recent studies have studied the clinical significance of NTR in different tumors, including esophageal cancer, breast cancer, and lung cancer, and explored the role of NTR in the assessment of lymph node metastasis. Cerfolio et al. [18, 19] reported that an NTR of 0.56 is the best cut-off value for predicting mediastinal lymph node metastasis in patients with non-small cell lung cancer, and its value is significantly better than that of the primary tumor SUVmax; Park et al. [20] also confirmed that in breast cancer, NTR could better predict metastasis to axillary lymph nodes than SUVmax in the lymph node with the highest axillary metabolism. Several studies have further explored the role of NTR in clinical prognosis. Kaira et al. [21] found that in non-small cell lung cancer, high NTR has a worse PFS and OS than a low NTR, and it did not respond well to initial therapy. Similarly, Kim HR [22], Kim YH [23], and Chung [24, 25] also confirmed that NTR is an independent influencing factor in predicting cervical squamous cell carcinoma, endometrial carcinoma, invasive ductal breast cancer, and resectable pancreatic cancer.

Currently, there is only one related report on the application of NTR in nasopharyngeal carcinoma, which showed the prognostic value of NTR in nasopharyngeal carcinoma DMFS. Hung et al. [6] showed that NTR is an independent influencing factor of DMFS in nasopharyngeal carcinoma. However, there was only one survival endpoint of DMFS that was analyzed, and there are no relevant reports on the prognosis of other survival endpoints. Our study is the first to report the value of NTR in OS and LPFS of nasopharyngeal carcinoma. Chen [1] mentioned that induction chemotherapy before concurrent radiotherapy and chemotherapy is a good treatment strategy because this method can improve the survival of patients with nasopharyngeal carcinoma and the control rate of distant metastasis. However, the eligibility of patients for induction chemotherapy needs further research. In the present study, we found that the 3-year OS, 3-year LPFS, and 3-year DMFS of patients with NTR < 0.95 were higher than those with NTR > 0.945 (*P* = 0.001, *P* = 0.045, and *P* = 0.032), and NTR was the independent factor for predicting OS and LPFS, suggesting that the metabolic activity of metastatic lymph node SUVmax relative to the primary tumor might be a promising prognostic indicator for patients with nasopharyngeal carcinoma. Moreover, our study also found that NTR was an independent influencing factor of DMFS in nasopharyngeal carcinoma, which was consistent with the results of Hung et al. [6]. This conclusion also suggested that in future work, we should pay attention to the relative metabolic activity of lymph nodes and primary tumors. For those with a high ratio of these two, appropriate additional radiation doses could be added during treatment to increase the tumor LPRS rate and prolong the patient’s OS, reducing the rate of distant metastasis. As this study aimed to investigate whether the relative metabolic activity of lymph nodes and primary tumors had prognostic value in locally advanced nasopharyngeal carcinoma, we found that NTR obtained by preprocessing was an independent predictor of OS and LC. Therefore, we believe that NTR might become a valuable prognostic indicator, allowing patients to benefit from more aggressive treatment. Our univariate analysis showed that LN-SUVmax was an important factor in predicting recurrence, while multivariate analysis did not. The non-standardized SUV used in different studies might be the reason for this difference. Moreover, the patient’s tumor type, blood sugar level, the interval between imaging agent injection time and scanning time, machine type, collection time per bed, and other factors could affect the scanning effect, leading to SUV variability. Different from previous studies, our study added another parameter NTR, the ratio of SUVmax of lymph node metastases to SUVmax of the primary tumor, to predict OS, LPFS, and DMFS of advanced nasopharyngeal carcinoma. NTR could also reflect the biological interaction between the primary tumor of nasopharyngeal carcinoma and metastatic lymph nodes. A recent study by Park et al. [20] showed that in breast cancer, the ratio of SUV of axillary metastatic lymph nodes to the SUV of the primary tumor is more accurate than the SUVmax of axillary metastatic lymph nodes to determine the rate of lymph node metastasis involvement.

The main limitation of this study was that it was a retrospective study conducted in a single center. Different chemotherapy regimens could affect the results. Furthermore, although our hospital had a standardized plan for PET scanning to minimize possible variations between scanners, biological and technical variations would still exist, which might lead to selection bias, thereby limiting the universality of our findings. Further verification of the prognostic value requires prospective and large-scale research on NTR before it can be widely used in clinical practice. Finally, we did not obtain lymph node pathology, and nasopharyngeal carcinoma differs from other head and neck cancers in that it is often treated in the absence of the pathological analysis of metastatic lymph nodes. It may be impossible to obtain the pathology of cervical lymph node metastasis due to ethical reasons. There have been studies [26] confirming that cervical lymph node puncture or biopsy can promote the occurrence of distant metastasis. Therefore, all cases in this study were diagnosed by the biopsy of the primary lesion in the nasopharynx with the nasopharyngoscope, while lymph node metastasis was determined according to the imaging diagnostic criteria. Nevertheless, this study is still worthy of attention because it is the first study to report NTR in the prognosis of locally advanced nasopharyngeal carcinoma. Our findings may serve as a reference for further research on the importance of metabolic activity related to metastatic lymphatic pretreatment.

## Conclusions

In summary, high NTR before treatment indicates poor prognosis in patients with nasopharyngeal carcinoma. This can serve as a reference value for the reasonable treatment and monitoring of prognosis in such patients.

## Data Availability

The datasets generated during and/or analyzed during the current study are available from the corresponding author on reasonable request.

## Abbreviations

NPC: Nasopharyngeal carcinoma
SUV: Maximum standardized uptake value
LN-SUVmax: Lymph node metastases SUVmax
LN-MTV: MTV of lymph node metastases
LN-TLG: TLG of lymph node metastases
T-SUVmax: Primary tumor SUVmax
T-MTV: MTV of primary nasopharyngeal carcinoma
T-TLG: TLG of primary nasopharyngeal carcinoma
NTR: The ratio of LN-SUVmax to T-SUVmx
ROC: Analysis receiver operating characteristic analysis;
AUC: Area-under-curve
CI: Confidence interval
CRT: Chemoradio therapy
OS: Overall survival
DMFS: Distant metastasis-free survival
LPFS: Local progression -free survival
FDG PET/CT: 2-Deoxy-2-^18^F fluoro-d-glucose positron emission tomography/computed tomography
HR: Hazard ratio
AJCC: American Joint Committee on Cancer
UICC: Union for International Committee on Cancer
IGRT: Image Guided Radiation Therapy
VMAT: Volumetric Modulated ArcTherapy
MTV: Metabolic tumor volume
TLG: Total lesion glycolysis
PFS: Progression-free survival
